# The optimal threshold of serum ceruloplasmin in the diagnosis of Wilson’s disease: A large hospital-based study

**DOI:** 10.1371/journal.pone.0190887

**Published:** 2018-01-11

**Authors:** Rong Xu, Yong-fang Jiang, Yong-hong Zhang, Xu Yang

**Affiliations:** 1 Institute of Metabolism and Endocrinology, The Second Xiangya Hospital, Central South University, Changsha, China; 2 Department of Infectious Diseases/ Liver Disease Research Center, The Second Xiangya Hospital, Central South University, Changsha, China; The Institute of Cognitive Sciences and Technologies, ITALY

## Abstract

**Background and aims:**

A ceruloplasmin (CP) concentration <200 mg/L is conventionally considered as one of the major diagnostic criteria for Wilson’s disease (WD). However, the diagnostic accuracy of this threshold has never been investigated in a sufficiently large group of patients. This study aims to present the results of serum CP measurements in various patients and to identify the optimal cutoff value of CP for the diagnosis of WD.

**Materials and methods:**

We identified patients whose CP levels were evaluated from January 1, 2016 to December 31, 2016 using a laboratory information database. Data related to CP measurement were retrieved. We carefully reviewed patients’ electronic medical records to correct errors and to obtain other necessary data. Data related to WD were retrieved from a special document containing medical records of patients with WD, which were created, modified, and maintained by authors.

**Results:**

CP level was determined in 4048 patients (WD, 297; non-WD, 3751). The mean serum CP level in patients with WD was 50.6±44.2 mg/L, which was significantly lower than that in non-WD patients (293.2±117.3 mg/L, p<0.001). Only 1.0% of patients with WD had CP ≥200 mg/L. The sensitivity and specificity of CP for the diagnosis of WD were 99.0 and 80.9%, respectively, for the conventional cutoff value <200 mg/L and 95.6 and 95.5%, respectively, for the cutoff value <150 mg/L; the latter provided a higher diagnostic accuracy for WD. 53.0% of patients with liver failure, 37.7% of patients with nephrotic syndrome, and 23.0% of patients age 1 to 6 months had serum CP <200 mg/L. Patients who were pregnant and those with malignant tumors, and infectious and inflammatory diseases had significantly higher mean serum CP levels.

**Conclusion:**

The optimal cutoff value of CP for the diagnosis of WD in China is 150 mg/L, with a sensitivity of 95.6% and specificity of 95.5%, thereby providing the highest diagnostic accuracy for WD.

## Introduction

Ceruloplasmin (CP) was first isolated from plasma and characterized by Holmberg and Laurell in 1948 as a copper-containing protein.[1] Soon thereafter, Scheinberg and Gitlin demonstrated a marked decrease in the concentration of this protein in serum samples from patients with Wilson’s disease (WD).[2] Serum CP level measurement is the first step in screening for WD. A CP concentration <200 mg/L is commonly considered one of the main diagnostic criteria for WD.[3] However, the diagnostic accuracy of this threshold has never been investigated or validated in a sufficiently large group of patients with and without WD. Furthermore, most of the available data on CP threshold for the diagnosis of WD are primarily from Europe and USA. Racial differences in CP possibly exist, which may in turn limit the generalizability of data to other regions. Two lower thresholds have been suggested by authors from Hong Kong [4] and Korea,[5] i.e., 140 mg/L and 160 mg/L, respectively. However, the study from Hong Kong had an extremely small sample size (57 patients with WD and 136 non-WD patients), and the study from Korea included only pediatric patients with hepatitis. Thus, further studies with a larger number of patients are necessary.

In clinical practice, several factors may affect the serum CP levels. Serum CP may be low in several conditions, including severe nephrotic syndrome and protein losing enteropathy, severe end-stage liver disease of various etiology, and severe copper deficiency, as well as certain neurological conditions.[6] Moreover, serum CP concentration is age-dependent.[7] Physiologically, serum CP levels are very low in normal neonates and elevate to adult levels by the age of 6 months, further increasing to a maximum concentration between 2–3 years old, (approximately 300–500 mg/L, which is higher than the adult level) and then gradually decreasing to the adult range until the teenage period. In addition, as an acute phase reactant, the serum CP concentration increases during inflammation, infection, and trauma, largely as a result of increased gene transcription in the hepatocytes, as mediated by inflammatory cytokines.[8] However, to the best of our knowledge, few prior studies on CP included an adequate number of patients and appropriate conditions. The conditions and the proportion of patients with abnormal CP levels remain unclear. This study aims to present the results of serum CP measurements in patients with different gender, age, and disorders and to identify the most appropriate cutoff value of CP for the diagnosis of WD in China.

## Materials and methods

### Study setting

This study was conducted in the Second Xiangya Hospital, Central South University, which is the largest and oldest university-affiliated hospital in Hunan Province, P. R. of China, with 4045 sickbeds and >3,000,000 outpatients per year. An advanced and modernized hospital information system has been implemented for several years in the hospital, including electronic patient databases and laboratory information databases; thus, a clinician can easily access clinical data depending on his or her role, needs, and security rights. This study conformed to the Helsinki Declaration (1975) and was approved by the Ethics Committee of the Second Xiangya Hospital, Central South University. Our hospital ethics committee waived the requirement for informed consent.

### Data sources

The main data source of this study was our hospital laboratory information database, which contains laboratory information of all patients tested in our hospital. We used the database to identify patients tested for CP from January 1, 2016 to December 31, 2016, and we retrieved all data related to CP measurement, including test results and time, patient’s name, sex, age, diagnosis, medical record number, physician’s name, and the department that ordered the test.

We corrected errors in patients’ demographics and diagnosis based on a comprehensive review of patients’ electronic medical records, which were similar to traditional paper charts and contained a problem list, pharmacy data, orders, laboratory results, progress notes, vital signs, radiology results, transferred record, and reports on various procedures, such as endoscopy.

Another data source was our special document of medical records of patients with WD, which we created, modified, and maintained.

### Study population and definitions

All patients who had their serum CP tested during the study period were eligible for the study. Outpatients whose data reliability could not be validated and inpatients whose data reliability could not be validated because of a wrong medical record number were excluded from the study.

Majority of the patients with WD were diagnosed by the authors. In addition to serum CP level, patients suspected of having WD underwent a slit-lamp examination for Kayser-Fleischer (K-F) rings, neurological examination, and determination of 24-h urinary copper excretion before and after penicillamine challenge. If not contraindicated, liver biopsy was performed. The WD gene mutation analysis was performed in some cases. The diagnosis of WD was based on typical neurological symptoms, positive K-F rings by slit lamp examination, low serum CP levels, elevated urinary copper excretion before and after penicillamine challenge, a hepatic copper content of ≥250 μg/dry weight in the absence of cholestasis, and disease mutations identified, as previously described.[3,9] The WD scores were calculated according to a slightly modified Leipzig scoring system published in 2003.[3,10] The diagnosis of WD was established on a WD score of four or more.

The WD phenotypes were classified according to previously published criteria.[10]

For few patients with WD diagnosed by other physicians, chart review was performed, the diagnosis of WD was based on firm evidence of confirmatory tests. All patients with WD had a WD score of four or more. The diagnoses of other various conditions were based on electronic medical record review, which employed the International Classification of Diseases, 10th Revision (ICD-10^th^), codes to identify specific clinical conditions.

### Laboratory methods

Routine laboratory data were obtained using standard methods. K-F rings were examined under a slit lamp by a single ophthalmologist with rich experience. Serum CP levels were measured using the nephelometric method (normal range, 210–500 mg/L; Beckman Coulter, Immage® Immunochemistry System, Brea, CA, USA). Copper levels in the serum, urine, and liver were determined using flame atomic absorption spectrophotometry at a wavelength of 324.7 nm, as previously described. [9,11] The *ATP7B* coding region and exon/intron boundaries were amplified using a polymerase chain reaction and sequenced in both directions, as previously described.[12]

### Statistical analysis

All statistical analyses were performed using SPSS (version 16; SPSS Inc., Chicago, IL, USA). Patient characteristics are presented as mean ± SD. The diagnostic accuracy of the test was assessed in terms of sensitivity, specificity, and positive and negative predictive value (PPV and NPV, respectively). Receiver operating characteristic (ROC) curve, which was used to determine the optimal cutoff value of serum CP level for the diagnosis of WD, was constructed using the data from patients with WD and those with other various diseases.

## Results

Between January 1, 2016 and December 31, 2016, CP was measured 7325 times in 6023 unique patients. Among them, 1935 outpatients, whose data reliability could not be confirmed, were excluded from the study. Forty inpatients whose data reliability could not be validated because of a wrong medical record number or wrong telephone number were also excluded from the study; the remaining 4048 patients were included.

### Serum ceruloplasmin levels in patients with Wilson’s disease

A total of 297 patients had WD, of which 53 patients were newly diagnosed (50 patients were diagnosed by authors) and 244 patients were diagnosed before 2016 (230 patients were diagnosed and followed up by authors). The mean age of the patients at diagnosis was 21.8±12.9 years (range, 2–62 years). The mean serum CP level in the patients diagnosed in 2016 was similar to that in the patients diagnosed before 2016. The mean serum CP level in the 297 patients was 50.6±44.2 mg/L, which was significantly lower than that in the non-WD patients (p<0.001). CP <200 mg/L was noted in 99.0% of patients with WD, and 84.8% of patients with WD had CP <100 mg/L. Only three patients (1.0%) had CP ≥200 mg/L (Table 1).

**Table 1:** Serum ceruloplasmin concentrations in patients with WD.

Time of diagnosis	Mean age (years)	Ceruloplasmin concentration (mg/L)					
		0-	50-	100-	150-	≥200	Mean
In 2016 (n = 53)	20.8±11.6	39(73.2%)	7(13.2%)	6(11.3%)	0	1(1.9%)	50.7±44.8
Before 2016 (n = 244)	22.0±12.0	170(69.7%)	36(14.8%)	26(10.7%)	10(4.1)	2((0.8%)	50.4±44.2
Total (n = 297)	21.8±11.9	209(70.4%)	43(14.5%)	32(10.8%)	10(3.4%)	3(1.0%)	50.6±44.3

### Serum ceruloplasmin levels in patients with other conditions

CP was measured in 3751 unique patients with various diseases other than WD (male, 2383; female, 1368) (Table 2). The mean age of the patients during the test was 29.6±22.9 years (range, 1 month-86 years). The mean serum CP level in these patients was 293.2±117.3 mg/L, which was significantly higher than that in patients with WD (p<0.001). Patients with acute and subacute, chronic liver failure had the lowest mean serum CP (200.8±55.3 mg/L, 218.6±73.8 mg/L, respectively); 53.0% of patients with acute and subacute liver failure had CP <200 mg/L and 12.6% had CP <150 mg/L. However, only 0.9% of patients had CP <100 mg/L. 3 patients with chronic liver failure had CP <100 mg/L, the possibility of WD had been excluded in all of them through subsequent clinical follow-up. Patients with nephrotic syndrome had very low mean serum CP (235.4±88.3 mg/L); 37.7% of patients had CP <200 mg/L, 13.9% had CP <150 mg/L, 2.2% had CP <100 mg/L, and two patients had CP <50 mg/L and the possibility of WD had been excluded in these two patients through subsequent clinical follow-up too. Patients with other liver disease and nervous and mental diseases also had significantly lower mean serum CP levels. Moreover, pregnant women had the highest mean serum CP (623.4±218.5 mg/L). Other conditions with significantly higher mean serum CP levels included malignant tumor, leukemia, various infectious diseases, mucocutaneous lymph node, hyperthyroidism, and connective tissue diseases.

**Table 2:** Serum ceruloplasmin levels in patients with various diseases.

Classification of diseases	Ceruloplasmin level (mg/L)					
	Mean	0-	50-	100-	150-	≥200
Acute hepatitis (n **=** 62)	378.6±115.9	0	0	0	0	62
Chronic hepatitis (n **=** 228)	249.3±88.2	0	0	9	53	166
Cirrhosis (n = 490)	260.9±85.0	0	0	25	101	364
Acute and subacute liver failure (n = 162)	200.8±55.3	0	0	23	72	67
Chronic liver failure (n = 183)	218.6±73.8	0	3	16	69	95
Drug-induced hepatitis (n = 60)	340.9±113.1	0	0	1	4	55
Autoimmune hepatitis (n = 44)	321.2±119.5	0	0	0	2	42
Primary biliary cirrhosis (n = 44)	360.8±120.5	0	0	0	4	40
Alcoholic liver disease and NAFLD (n = 142)	283.4±81.3	0	0	7	16	119
Other digestive tract diseases (n = 129)	307.0±107.5	0	0	5	11	113
Nephrotic syndrome (n = 324)	235.4±88.3	2	5	38	77	202
Purpura nephritis (n = 120)	263.1±77.9	0	0	6	18	96
Other kidney disease (n = 160)	283.2±75.3	0	0	2	16	142
Parkinsonism (n = 16)	281.9±60.8	0	0	0	2	14
Schizophrenia (n = 28)	262.7±73.7	0	0	1	3	24
Depression (n = 12)	254.3±72.6	0	0	0	1	11
Epilepsy (n = 103)	278.2±69.6	0	0	1	8	94
Autonomic nervous dysfunction (n = 28)	263.9±68.0	0	0	0	3	25
Myasthenia gravis (n = 64)	283.1±62.0	0	0	0	3	61
Other nervous, mental disorders (n = 167)	275.8±67.0	0	0	4	16	147
Purpura (n = 156)	294.7±78.8	0	0	4	13	139
Connective tissue diseases (n = 124)	353.2±140.7	0	0	2	8	114
Mucocutaneous lymph node (n = 11)	452.3±155.7	0	0	0	0	11
Cardiovascular disease (n = 77)	304.0±101.4	0	0	3	8	66
Hyperthyroidism (n = 32)	375.3±156.2	0	0	0	1	31
Diabetes mellitus (n = 27)	286.9±81.5	0	0	0	3	24
Sepsis (n = 81)	381.2±119.0	0	0	0	3	78
Pneumonitis and bronchiolitis (n = 158)	338.8±91.7	0	0	1	6	151
Infectious mononucleosis (n = 15)	415.5±86.5	0	0	0	0	15
Intracranial infection (n = 121)	314.6±104.6	0	0	0	7	114
Pregnancy (n = 47)	623.4±218.5	0	0	0	0	47
Malignant tumor and leukemia (n = 229)	392.1±152.5	0	1	2	12	214
Other diseases (n = 107)	326.8±124.0	0	2	2	13	90
Total (n = 3751)	293.2±117.3	2	11	152	553	3033

NAFLD; nonalcoholic fatty liver disease

### Serum ceruloplasmin levels in different age groups

The mean serum CP levels in 3751 non-WD patients stratified by age groups are presented in Table 3. Patients age 1 to 5 months had the lowest mean serum CP level (262.0±119.5 mg/L). Among them, 23.0% of patients had serum CP <200 mg/L and 8.1% had serum CP <150 mg/L; however, no patient had serum CP <100 mg/L. The value rapidly increased to adult concentration by the age of 1 year and further increased to the maximum concentration by the age of 1 to 5 years. Thereafter, the value gradually decreased back to the adult concentration during the teenage years. The concentration increased again by the age of 60 years, and the second peak of concentration was noted in patients >70 years of age.

**Table 3:** Serum ceruloplasmin concentrations in patients stratified by age.

Age group	No. of patients	Ceruloplasmin concentration (mg/L)					
		Mean	0-	50-	100-	150-	≥200
1 month-	74	262.0±119.5	0	0	6	11	57
6 months-	17	274.9±92.0	0	0	0	2	15
1 year-	462	327.4±113.5	0	1	8	26	427
6 years-	644	302.9±106.2	1	2	19	68	554
10 years-	553	267.0±99.6	0	4	33	90	426
20 years-	209	307.3±172.4	1	1	13	39	155
30 years-	307	277.4±142.1	0	2	14	81	210
40 years-	518	266.4±103.3	0	1	35	109	373
50 years-	505	297.8±112.7	0	0	11	75	419
60 years-	344	306.0±108.1	0	0	11	43	290
70 years-	118	329.3±120.0	0	0	2	9	107
Total	3751	293.2±117.3	2	11	152	553	3033

### Diagnostic accuracy of ceruloplasmin for Wilson’s disease

**Fig 1:**
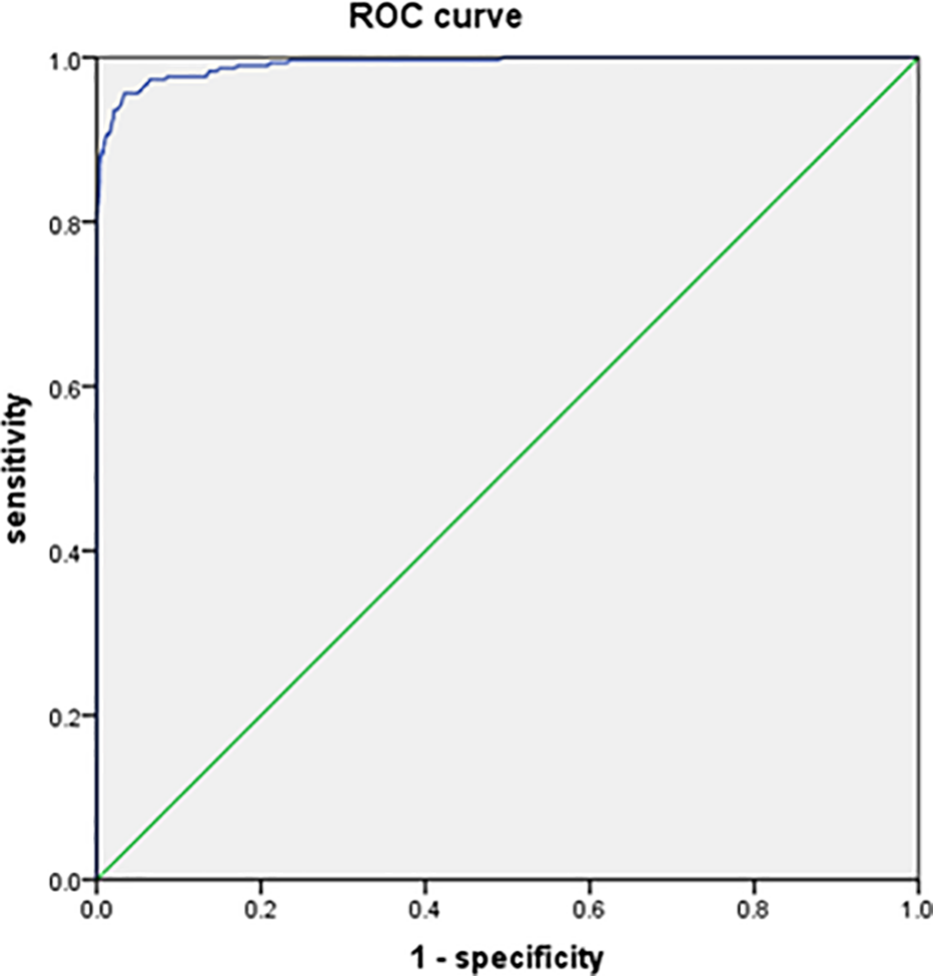
ROC curve of serum ceruloplasmin concentration for the diagnosis of WD. The curve was constructed using the data of 297 patients with WD and 3751 non-WD patients. The area under the curve was 0.992 (95% confidence interval (CI), 0.987–0.996).

The sensitivity, specificity, PPV, and NPV of serum CP level for the diagnosis of WD at the conventional cutoff value <200 mg/L were 99.0, 80.9, 29.1, and 99.9%, respectively, in the whole group and 99.0, 76.4, 38.9, and 99.8%, respectively, in patients with liver, nervous, and mental diseases (Tables 4 and 5). An ROC curve of serum CP level for the diagnosis of WD was constructed using the data of 297 patients with WD and 3751 non-WD patients (Fig 1). The area under the curve was 0.992 (95% confidence interval (CI), 0.987–0.996). The ROC curve suggested that the most useful cutoff value is 150 mg/L, with a sensitivity of 95.6% and specificity of 95.5%, thereby providing the highest diagnostic accuracy for WD. Another ROC curve of serum CP level was constructed using the data of 297 patients with WD and 1962 patients with liver, nervous, and mental diseases (the figure was omitted). The area under the curve was 0.991 (95% CI, 0.986–0.996). Similarly, the ROC curve suggested that the most useful cutoff value is 150 mg/L, with a sensitivity of 95.6% and specificity of 95.0%, thereby also providing the highest diagnostic accuracy for WD.

**Table 4:** Diagnostic accuracy of ceruloplasmin at the conventional cutoff value in whole group.

CP(mg/L)	No. of patients diagnosed			Sensitivity(%, 95% CI)	Specificity(%, 95% CI)	PPV(%, 95% CI)	NPV(%, 95% CI)
	WD	Non-WD	Total				
< 200	294	718	1023	99.0(98.9–99.1)	80.9(79.6–82.2)	29.1(26.3–31.9)	99.9(99,8–100.0)
≥ 200	3	3033	3036				
Total	297	3751	4048				

CP, CI, confidence interval, Ceruloplasmin; WD, Wilson’s disease; PPV, positive predictive value; NPV, negative predictive value.

**Table 5:** Diagnostic accuracy of ceruloplasmin at the conventional cutoff value in patients with liver, nervous, and mental diseases.

CP(mg/L)	No. of patients diagnosed			Sensitivity(%, 95% CI)	Specificity(%, 95% CI)	PPV(%, 95% CI)	NPV(%, 95% CI)
	WD	Non-WD	Total				
< 200	294	463	757	99.0(98.9–99.1)	76.4(74.5–78.3)	38.8(35.3–42.3)	99.8(99,7–99.9)
≥ 200	3	1499	1502				
Total	297	1962	2259				

CP, CI, confidence interval, Ceruloplasmin; WD, Wilson’s disease; PPV, positive predictive value; NPV, negative predictive value.

## Discussion

A CP concentration <200 mg/L is often considered the major cutoff for the diagnosis of WD.[3] However, this threshold is mainly based on data from Europe and the US, and its rationality and usefulness have never been validated in a sufficiently large group of patients in China. Given the possible regional differences in CP concentration, we investigated the diagnostic accuracy of CP for WD in China. Our study showed that the conventional cutoff value <200 mg/L resulted in a sensitivity of 99.0%, which in turn led to unacceptably high false-positive rates (whole group, 19.1%; patients with liver, nervous, and mental conditions, 23.6%). The ROC curves suggested that the most useful cutoff value is 150 mg/L in both groups, where sensitivity was 95.6%, and false positive-rates were 5.0% (whole group) and 4.8% (patients with other conditions), thereby providing the highest diagnostic accuracy for WD. These results were consistent with those of previous studies from Hong Kong and Korea.[4–5]

In discussing the threshold, two other aspects should be considered. First, the selection of the optimal cutoff values for a test by means of the Youden index may lead to an overestimation of sensitivity and specificity. The amount of bias in sensitivity and specificity predominantly depends on the sample size.[13] As our study had an extremely large sample size, the amount of bias in sensitivity and specificity caused by the sample size would be expected to be negligible. Second, the choice of the threshold of a test depends not only on its accuracy but also on disease prevalence and clinical benefits and costs, which could be associated with correct and incorrect subject classification.[14–15] For a screening test of a very low-prevalence disease such as WD, a low threshold should be chosen to favor specificity. When the threshold of 150 mg/L was used, the sensitivity decreased by only 3.4%, and the false-positive rate decreased by 14.1%, which in turn prevented 553 false-positive patients (150 mg/L>CP<200 mg/L) from undergoing unnecessary further investigations. Therefore, the new threshold may be more cost-effective than the conventional one.

One important finding of this study was that very few patients with WD had normal CP levels in our study. Most reports indicated that 90–100% of patients with WD had serum CP in the subnormal range.[16–17] However, an extremely high proportion of patients with WD with normal CP levels has been reported by numerous authors. In one series, 12 of 55 patients with WD had normal CP levels, [18] and in another study, six of 22 patients with WD also had normal CP levels.[19] In a previous study, 10 of 28 children with WD had serum CP >200 mg/L.[20] By contrast, very few patients with WD (1%) had normal CP levels in our study. Although the CP level can vary based on different measurement methods, standardization, and subject age, the discrepancy between our findings and those of previous studies could not be explained by these factors because CP was measured using standard reagents and because we included all age groups in our study. Through literature review, we found that the proportion of patients with WD with normal CP levels had obvious regional differences, and few patients with WD from China and Korea had normal CP levels. In a study reported by Lee BH et al. from Korea, [21] none of 237 patients with WD had normal CP levels. In a study reported by Feng L et al. from China, [22] none of 126 patients with WD had normal CP levels, and in another study reported by Liu Y et al. from China,[23] one of 57 patients with WD had normal CP levels [Table 6]. Therefore, we speculated that some unclear regional factors from China and Korea contributed to this discrepancy.

**Table 6:** Proportion of patients with WD with normal ceruloplasmin levels in different regions.

Author	Country/time	Patients with Wilson’s disease	
		Total number of patients	Patients with normal CP levels
Brewer GJ et al. [24]	USA/1992	89	8(11.2%)
Martins da Costa C [25]	United Kingdom/1992	17	3(17.6%)
Steindl P et al. [18]	Germany/1997	55	15(27.3%)
Merle U et al. [26]	Germany/2007	163	19(11.8))
Gheorghe L et al. [27]	Romania/2004	55	24(43.6%)
Müller T et al. [28]	Austria/2007	35	9(25.7%)
Manolaki N et al. [29]	Greece/2008	57	7(12.3%)
Nicastro E et al. [30]	Italy/2010	40	2(5.0%)
El-Karaksy H et al. [31]	Egypt/2011	54	1(1.9%)
Taly AB et al. [32]	India/2007	282	19(6.9%)
Rukunuzzaman MD [33]	Bangladesh/2015	100	27(27.0%)
Lee BH et al. [21]	Korea/2011	237	0(0.0%)
Mak CM et al. [4]	Hong Kong/2008	57	1(1.8%)
Liu Y et al. [23]	China/2015	77	2(2.6%)
Feng L et al. [22]	China/2016	126	0(0.0%)
This study	China/2017	297	3(1.0%)

Another important finding of this study was that as many as 19.1% of non-WD patients had CP <200 mg/L; however, the CP level was only slightly decreased in most of them, and only 4.4% had CP <150 mg/L, and 0.3% had CP <100 mg/L. It is well known that CP levels decrease in some patients with the nephrotic syndrome, and it has been suggested that the low CP levels can result from CP loss in the urine. [34] However, CP levels have never been investigated in a large cohort of patients with nephrotic syndrome. Our study confirmed that the mean CP level is significantly lower in patients with nephrotic syndrome than in those with other conditions. Of 324 patients with nephrotic syndrome, 37.7% of patients had CP <200 mg/L, 13.9% had CP <150 mg/L, and 2.2% had CP <100 mg/L. A CP level as low as 20 mg/L was noted in two patients. These results suggest that nephrotic syndrome should be considered in the differential diagnosis of patients with extremely low CP levels. Among all non-WD patients, those with acute and subacute liver failure had the lowest mean serum CP (200.8±55.3 mg/L). Of 345 patients with liver failure (acute, subacute, and chronic), 53.0% of patients had CP <200 mg/L, and 12.1% of patients had CP <150 mg/L; however, unlike in nephrotic syndrome, only a few patients had CP <100 mg/L, and no patient had CP <50 mg/L. Although patients with liver failure may have a low CP level, [35] as many as 53.0% of patients with liver failure with CP <200 mg/L have not been reported. As severe overlap in CP concentration between WD and non-WD liver failure exists, WD could not be confirmed or excluded according to the CP concentration alone.

Several studies have examined the nature, functions, and regulation of plasma CP.[36–37] However, in actual clinical practice, few studies have measured the CP levels in a large number of patients with various conditions. Our study with 297 patients with WD and 3751 non-WD patients, including different age groups and different conditions, is currently the largest study on CP. In this study, pregnant women were found to have the highest mean CP level (623.4±218.5 mg/L), which was significantly higher than those in other groups. Among 47 pregnant women, no patient had subnormal CP levels. It is well established that estrogen and progesterone have enhancing effects on the expression of CP in the blood plasma. The levels of CP increase during pregnancy and in women taking oral contraceptives.[38] Our results were consistent with those of previous studies. In addition, it is well known that CP is an acute phase reactant, and CP levels in the blood plasma increase when the immune system responds to infection and inflammation. Inflammatory responses are largely mediated by cytokines.[39–40] Our study has shown that patients with malignant tumors, leukemia, various infectious diseases, mucocutaneous lymph node, hyperthyroidism, and connective tissue diseases had significantly higher mean serum CP levels, and few of these patients had subnormal CP levels. These results were consistent with those of previous studies.

In conclusion, very few patients with WD (1%) had normal CP levels in China, and 19.1% of non-WD patients and 53.0% of patients with acute and subacute liver failure had CP <200 mg/L. The most useful cutoff value of CP for the diagnosis of WD is 150 mg/L, which provides the highest accuracy for the diagnosis of WD.

## References

[1] Holmberg CG, Laurell CB. Investigations in serum copper. II. Isolation of the copper-containing protein and a description of some of its properties. Acta Chem. Scand. 1948; 2:550–556. PMID:8876909

[2] Scheinberg IH, Gitlin D. Deficiency of ceruloplasmin in patients with hepatolenticular degeneration (Wilson’s disease). Science 1952; 31;116 (3018):484–485. 12994898 10.1126/science.116.3018.484

[3] Roberts EA, Schilsky ML. Diagnosis and treatment of Wilson disease: an update. Hepatology 2008; 47(6): 2089–2111. https://doi.org/10.1002/hep.22261 18506894 10.1002/hep.22261

[4] Mak CM, Lam CM, Tam S. Diagnostic accuracy of serum ceruloplasmin in Wilson Disease: determination of sensitivity and specificity by ROC curve analysis among ATP7B-genotyped subjects. Clinical Chemistry 2008; 54:8:1356–1362.https://doi.org/10.1373/clinchem.2008.103432 18556333 10.1373/clinchem.2008.103432

[5] Kim JA, Kim HJ, Cho JM, Oh SH, Lee BH, Kim GH, et al Diagnostic value of ceruloplamin in the diagnosis of pediatric Wilson’s disease. Pediatr Gastroenterol Hepatol Nutr 2015;18(3):187–92. https://doi.org/.10.5223/pghn.2015.18.3.187 26473139 10.5223/pghn.2015.18.3.187 PMC4600703

[6] Walshe JM. Diagnostic significance of reduced serum caeruloplasmin concentration in neurological disease. Mov Disord 2005;20:1658–1661. doi: 10.1002/mds.20628 16092117 10.1002/mds.20628

[7] Cox DW. Factors influencing serum ceruloplasmin levels in normal individuals. J Lab Clin Med 1966; 68:893–904. 5926186

[8] Gitlin JD. Transcriptional regulation of ceruloplasmin gene expression during inflammation. J. Biol. Chem 1988; 263: 6281–6287. 3360784

[9] Yang X, Tang XP, Zhang YH, Luo KZ, Jiang YF, Luo HY, et al Prospective evaluation of the diagnostic accuracy of hepatic copper content, as determined using the entire core of a liver biopsy sample. Hepatology 2015; 62(6):1731–1741. https://doi.org/10.1002/hep.27932. 26095812 10.1002/hep.27932 PMC4744736

[10] Ferenci P, Caca K, Loudianos G, Mieli-Vergani G, Tanner S, Sternlieb I, et al Diagnosis and phenotypic classification of Wilson disease. Liver Int 2003;23:139–142. 12955875 10.1034/j.1600-0676.2003.00824.x

[11] Yang X, Tang XP, Zhang YH, Luo KZ, Jiang YF, Luo HY, et al Reply. Hepatology 2016; 64(4):1382–1383. https://doi.org/10.1002/hep.28565 26997619 10.1002/hep.28565 PMC5074240

[12] He G, Yang X, Luo KZ. A study of the liver pathology and direct sequencing of all exons of WD gene in a patient with fulminate Wilson disease. Chin J of Hepatol 2007;15:712–713. 17903386

[13] Leeflang MM, Moons KG, Reitsma JB, Zwinderman AH. Bias in sensitivity and specificity caused by data-driven selection of optimal cutoff values: mechanisms, magnitude, and solutions. Clinical Chemistry 2008; 54(4): 729–737. https://doi.org/10.1373/clinchem.2007.096032 18258670 10.1373/clinchem.2007.096032

[14] Sox HC, Blatt MA, Higgins MC, Marton KI. Medical decision making. Stoneham: Butterworths; 1988.

[15] Jund J, Rabilloud M, Wallon M, Ecochard R: Methods to estimate the optimal threshold for normally or log-normally distributed biological tests. Med Dec Making 2005; 5:406–415. https://doi.org/10.1177/0272989X05276855 PMID:1601892 10.1177/0272989X05276855 16061892

[16] Scheinberg IH, Sternlieb I. Wilson’s Disease. Philadelphia, PA: W B Saunders; 1984.

[17] Walshe JM. Wilson’s disease presenting with features of hepatic dysfunction: a clinical analysis of eighty-seven patients. Q J Med 1989; 70:253–263. 2602537

[18] Steindl P, Ferenci P, Dienes HP, Grimm G, Pabinger I, Madl C, et al Wilson’s disease in patients presenting with liver disease: a diagnostic challenge. Gastroenterology 1997;113:212–218. 9207280 10.1016/s0016-5085(97)70097-0

[19] Gow PJ, Smallwood RA, Angus PW, Smith AL, Wall AJ, Sewell RB. Diagnosis of Wilson’s disease: an experience over three decades. Gut 2000;46:415–419. doi: 10.1136/gut.46.3.415 10673307 10.1136/gut.46.3.415 PMC1727845

[20] Perman JA, Werlin SL, Grand RJ, Watkins JB. Laboratory measures of copper metabolism in the differentiation of chronic active hepatitis and Wilson disease in children. J Pediatr 1979;94:564–568. 430291 10.1016/s0022-3476(79)80011-6

[21] Lee BH, Kim JH, Lee SY, Jin HY, Kim KJ, Lee JJ. Distinct clinical courses according to presenting phenotype sand their correlations to ATP7B mutations in a large Wilson’s disease cohort. Liver International 2011; 6:831–9. https://doi.org/.10.1111/j.1478-3231.2011.02503.x PMID:21645214 10.1111/j.1478-3231.2011.02503.x 21645214

[22] Feng L, Wen MY, Wang WQ, Fan XL, Yang L. Clinical characteristics of Wilson's disease: a retrospective analysis of admission data among 126 Patients. Sichuan Da Xue Xue Bao Yi Xue Band 2016; 47(1):128–130. PMID:15702782 27062797

[23] Liu Y, Zhou H, Guo H, Bai Y. Genetic and clinical analysis in a cohort of patients with Wilson’s disease in Southwestern China. Archives of Medical Research 2015;46:164–169.https://doi.org/10.1016/j.arcmed.2015.02.001 25704634 10.1016/j.arcmed.2015.02.001

[24] Brewer FJ, Yuzbasiyan-Gurkan V. Wilson disease. Medicine (Baltimore) 1992; 71(3):139–164.PMID:1635139 1635439 10.1097/00005792-199205000-00004

[25] Martins da Costa C, Baldwin D, Portmann B, Lolin Y, Mowat AP, Mieli-Vergani G.Value of urinary copper excretion after penicillamine challenge in the diagnosis of Wilson’s disease. Hepatology 1992;15(4):609–615. 1551638 10.1002/hep.1840150410

[26] Merle U, Schaefer M, Ferenci P, Stremmel W. Clinical presentation, diagnosis and long-term outcome of Wilson’s disease: a cohort study. Gut 2007;56:115–120. doi: 10.1136/gut.2005.087262 16709660 10.1136/gut.2005.087262 PMC1856673

[27] Gheorghe L, Popescu I, Iacob S, Gheorghe C, Vaidan R, Constantinescu A, et al Wilson's Disease: a challenge of diagnosis. The 5-year experience of a tertiary centre. Rom J Gastroenterol. 2004; 13(3):179–185 15470529

[28] Müller T, Koppikar S, Taylor RM, Carragher F, Schlenck B, Heinz-Erian P, et al Re-evaluation of the penicillamine challenge test in the diagnosis of Wilson’s disease in children. J Hepatol 2007; 47(2):270–6. https://doi.org/10.1016/j.jhep.2007.03.011 17449133 10.1016/j.jhep.2007.03.011

[29] Manolaki N, Nikolopoulou G, Daikos GL, Panagiotakaki E, Tzetis M, Roma E, et al Wilson Disease in Children: Analysis of 57 Cases. J Pediatr Gastroenterol Nutr.2009;48(1):72–77 doi: 10.1097/MPG.0b013e31817d80b8 19172127 10.1097/MPG.0b013e31817d80b8

[30] Nicastro E, Ranucci G, Vajro P, Vegnente A, Iorio R. Re-evaluation of the diagnostic criteria for Wilson disease in children with mild liver disease. Hepatology 2010; 52(6):1948–56. doi: 10.1002/hep.23910 20967755 10.1002/hep.23910

[31] EI-Karaksy H, Fahmy M, EI-Raziky MS, EI-Hawary M, EI-Sayed R, EI-Koofy N, et al A clinical study of Wilson’s disease: The experience of a single Egyptian Paediatric Hepatology Unit. Arab Journal of Gastroenterology 2011; 125–130. doi: 10.1016/j.ajg.2011.07.007 22055589 10.1016/j.ajg.2011.07.007

[32] Taly AB, Meenakshi-Sundaram S, Sinha S, Swamy HS, Arunodaya GR. Wilson disease: description of 282 patients evaluated over 3 decades. Medicine (Baltimore) 2007; 86(2):112–121. doi: 10.1097/MD.0b013e318045a00e 17435591 10.1097/MD.0b013e318045a00e

[33] Rukunuzzaman MD. Wilson’s Disease in Bangladeshi Children: Analysis of 100 Cases of Wilson’s Disease in Bangladeshi Children. Pediatr Gastroenterol Hepatol Nutr 2015; 18(2):121–127 doi: 10.5223/pghn.2015.18.2.121 26157698 10.5223/pghn.2015.18.2.121 PMC4493245

[34] Mainero A, Cruz C, Pedraza-Chaverri J. Serum and urinary ceruloplasmin in experimental nephrotic syndrome. Clin Invest Med. 1992;15(4):295–300. 1516286

[35] Yang X, Tong DJ, Liang J, Zheng YH, Ler JH, et al Ceruloplasmin level of patients with liver disease in China. Zhonghua Nei Ke Za Zhi 2005; 44(1):13–15. Chinese. 15769390

[36] Bielli P, Calabrese L. Structure and function relationships in ceruloplasmin: a ‘moonlighting’ protein, Cell. Mol. Life Sci., 2002, 59, 1413–1427. 12440766 10.1007/s00018-002-8519-2 PMC11337412

[37] Gray LW, Kidane TZ, Nguyen A, Akagi S, Petrasek K, Chun YL, et al Copper proteins and ferroxidase in human plasma and that of wild-type and ceruloplasmin knochout mice. Biochem J. 2009 4 1;419(1):237–45. doi: 10.1042/BJ20081983 19076073 10.1042/BJ20081983

[38] Middleton RB, Linder MC. Synthesis and turnover of ceruloplasmin in rats treated with 17 beta-estradiol. Arch. Biochem. Biophys. 1993; 302: 362–368. doi: 10.1006/abbi.1993.1224 8489241 10.1006/abbi.1993.1224

[39] Mackiewicz A, Ganapathi MK, Schultz D, Samols D, Reese J, Kushner I. Regulation of rabbit acute phase protein biosynthesis, Biochem. J. 1988; 253: 851–857. 2460085 10.1042/bj2530851 PMC1149381

[40] Linder M. C. Ceruloplamin and other copper binding components of blood plasma and their functions: an update. Metallomics. 2016; 8(9):887–905. https://doi.org/10.1039/c6mt00103c. 27426697 10.1039/c6mt00103c

